# Gastrointestinal Mesenchymal Neoplasms other than Gastrointestinal Stromal Tumors: Focusing on Their Molecular Aspects

**DOI:** 10.4061/2011/952569

**Published:** 2011-02-16

**Authors:** Thomas P. Plesec

**Affiliations:** Cleveland Clinic, 9500 Euclid Avenue, L25, Cleveland, OH 44195, USA

## Abstract

Gastrointestinal (GI) mesenchymal tumors other than gastrointestinal stromal tumor (GIST) are rare neoplasms, but they often enter the differential diagnosis of more common GI lesions. Some of these mesenchymal tumors in the GI tract have well understood molecular pathologic aspects, including desmoid tumors, inflammatory myofibroblastic tumor (IMT), clear cell sarcoma (CCS), inflammatory fibroid polyp (IFP), and synovial sarcoma (SS). Molecular pathology is fast becoming a mainstream focus in laboratories because it aids in the precise classification of tumors, may be prognostic, and may help predict response to therapy. The following review is not intended as an exhaustive summary of all mesenchymal tumors that have been reported to involve the GI tract, but instead will highlight the current knowledge of the most important non-GIST GI mesenchymal neoplasms, focusing on those tumors with well-characterized molecular pathology and how the molecular pathologic features impact current diagnostic, therapeutic, and prognostic standards.

## 1. Introduction

Gastrointestinal (GI) mesenchymal tumors are rare, and the molecular pathology of many of these tumors is unknown or poorly characterized. However, some mesenchymal tumors in the GI tract have well-understood molecular pathologic aspects. Molecular pathology is fast becoming a mainstream focus in laboratories because it aids in the precise classification of tumors, may be prognostic, and may help predict response to therapy. A search of the catalogue of somatic mutations in cancer (COSMIC) database for all mesenchymal tumors in the tubular GI tract and adjacent soft tissues, including esophagus, stomach, small intestine, large intestine, peritoneum, and retroperitoneum reveals meaningful data on three tumor types: gastrointestinal stromal tumor (GIST), inflammatory fibroid polyp (IFP), and desmoid tumors. Other mesenchymal tumors that occur in or around the tubular GI tract with well-characterized molecular pathologic features include synovial sarcoma (SS), inflammatory myofibroblastic tumor (IMT), and clear cell sarcoma (CCS); these tumors are characterized by translocations rather than mutations. The following paper is not intended as an exhaustive summary of all mesenchymal tumors that have been reported to involve the GI tract, but instead will highlight the current knowledge of the most important non-GIST GI mesenchymal neoplasms, focusing on those tumors with well-characterized molecular pathology.

## 2. Intraabdominal Desmoid Tumors

**Figure 1:**
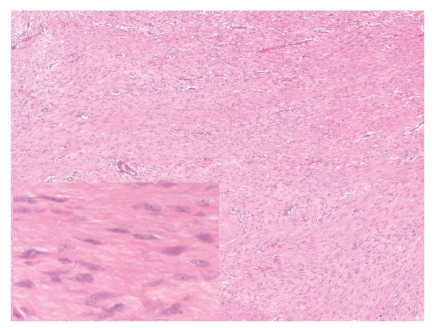
Photomicrograph of H&E-stained section from a desmoid tumor. Note the moderately cellular sweeping fascicles of bland spindle cells. The lower left inset contains a high-power photomicrograph of the same tumor to demonstrate the characteristic pinpoint nucleoli and collagenous stroma of desmoids.

Intraabdominal desmoid tumors arise in the mesentery or retroperitoneum, predominantly in young patients. Approximately 10% of desmoids occur in patients with familial adenomatous polyposis [1] as one of the extracolonic manifestations of Gardner syndrome. Desmoids do not metastasize, but they often recur locally [1, 2]. The histologic features of desmoids are quite characteristic. In particular, these tumors show low to moderate cellularity and are composed of uniform spindle cells with a small, distinct nucleolus arranged in long, sweeping fascicles (Figure 1) [1]. The vasculature shows small arteries with accompanying veins and a mild perivascular lymphoid infiltrate. The associated stroma is quite collagenous. Mitotic figures may be 10/50 high power field (HPF) or more [3, 4], but these tumors lack other histologic features of malignancy such as dense cellularity, cytologic atypia, or atypical mitotic figures. Important differential diagnostic considerations include sclerosing mesenteritis, which does not invade bowel wall [5] and IgG4-related sclerosing disorders, which are rich in IgG4 plasma cells [6].

**Figure 2:**
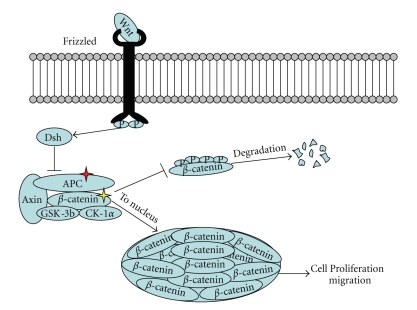
Schematic of Wnt signaling pathway. In demoid tumors, mutations are usually found in the *APC* gene or *CTNNB1* gene, which encodes *β*-catenin. Regardless of the primary defect, the end result is nuclear accumulation of *β*-catenin, which fails to undergo cytoplasmic degradation.

Most desmoid tumors arise via perturbations within the wnt signaling pathway (Figure 2). In FAP, desmoid tumors arise from mutations in the adenomatous polyposis coli (*APC*) gene, located on chromosome 5q21-22, which encodes a tumor suppressor protein, although its function may be more complex than simply a tumor suppressor [7]. Inactivation of *APC* leads to nuclear accumulation of *β*-catenin, causing increased transcription and cell proliferation [1]. Most *APC *mutations associated with desmoid tumors are found 3′ to codon 1400 [8, 9]. Some sporadic desmoid tumors also arise from *APC* inactivation [10] but most (>80%) are *APC* wild type with activating mutations of the *CTNNB1* gene, which is located on chromosome 3p22-p21.3. *CTNNB1 *encodes *β*-catenin [11]. Mutations almost exclusively occur at codons 41 and 45 in exon 3 of *CTNNB1 *[12]*; *mutational analysis of *CTNNB1* is usually not necessary in typical cases of desmoids tumors but can be helpful in unusual cases as well as helpful in distinguishing recurrent desmoid from scar. It remains to be determined whether particular *CTNNB1* mutations help predict local recurrence after surgical resection. One report [13] found that tumors harboring S45F mutations in exon 3 of *CTNNB1* had significantly poorer disease-free survival compared to wild-type tumors or codon 41 mutants, whereas another report showed no significant differences in recurrence-free survival among *CTNNB1* mutants but did show worse outcome among all mutants compared to wild-type tumors [14]. 

Regardless of inciting molecular event, the final common pathway is accumulation of *β*-catenin protein within the nuclei of the tumor cells, and although most desmoids do not require immunohistochemistry for diagnosis, nuclear staining of with *β*-catenin characterizes >90% of desmoid tumors [1]. In our experience, *β*-catenin immunohistochemistry is less reliable than the literature reports, particularly in needle biopsies, and molecular testing for mutations in the *β*-catenin gene may be more reliable. Immunoreactivity with CD117 has been reported in the literature [4], but this is generally considered an anomaly; when appropriate titers and antigen retrieval methods are used, desmoids show no CD117 positivity [15–18]. Although some investigators have reported response to imatinib [19], others have found that imatinib showed the lowest response rates in comparison to other forms of systemic therapy [20]. Furthermore, members of the PDGFR family are expressed in the majority of desmoids, but this expression does not correlate with response to imatinib. Since no clear target is present on desmoid tumors, imatinib therapy is controversial at best. Surgery remains the mainstay of treatment, but surgical resection is often incomplete because of the infiltrative growth of desmoid tumors, with recurrences up to 38% [13]. In these patients, systemic therapies such as anti-inflammatory, hormonal, cytotoxic chemotherapy, and radiation are considered. In patients with FAP, surgery such as ileal pouch procedures may trigger desmoid tumor formation [1].

## 3. Inflammatory Myofibroblastic Tumor (IMT)

**Figure 3:**
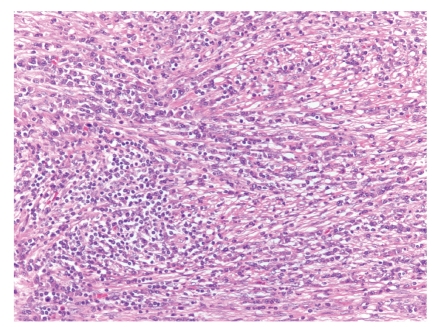
Photomicrograph of H&E-stained section from an inflammatory myofibroblastic tumor. The tumor is relatively cellular and composed of a mixture of plump spindle cells and inflammatory cells, particularly lymphocytes and plasma cells.

IMTs are a heterogenous group of spindle cell proliferations with admixed lymphocytes and plasma cells that tend to occur in children and young adults. The omentum and mesentery are the most common extra-pulmonary sites for IMT [21, 22], and these tumors may have a more aggressive biologic behavior, with more frequent recurrences [23]. Most IMTs harbor a heterogenous microscopic appearance and may contain any combination of nodular fasciitis-like areas, compact spindle cell proliferations, or paucicellular scar-like areas, making them a challenging diagnostic entity. In all tumors, inflammatory cells are a distinctive feature, and the infiltrate is often rich in plasma cells (Figure 3) [22].

Some authorities have differentiated IMTs from reactive pseudosarcomatous processes and other neoplasms based on ALK immunoreactivity or evidence of the *ALK* translocation, but many consider ALK-negative IMTs a valid diagnostic category. Tumors with *ALK* rearrangements are associated with younger age and strongly correlate with ALK protein expression detected by immunohistochemistry in some labs, but in other labs ALK immunohistochemistry is nearly always negative. Cook et al. found that 12/20 (60%) of cases expressed ALK by immunohistochemistry [24]. In fact, the literature reports that only about half of IMT harbor an *ALK* translocation, and these may behave more indolently than their ALK-negative counterparts [23], although this may not be the case for all *ALK*-rearranged tumors. For example, a very recent series described 11 intra-abdominal tumors characterized by epithelioid morphology, abundant myxoid stroma, and frequent neutrophils [25]. These tumors demonstrated aggressive behavior with rapid local recurrence, death in 5, and metastasis in 2. All 11 showed nuclear membrane or peri-nuclear ALK immunoreactivity. Nine tumors showed *ALK* translocation, and 3 tested showed *ALK/RANBP2* rearrangement. 

**Figure 4:**
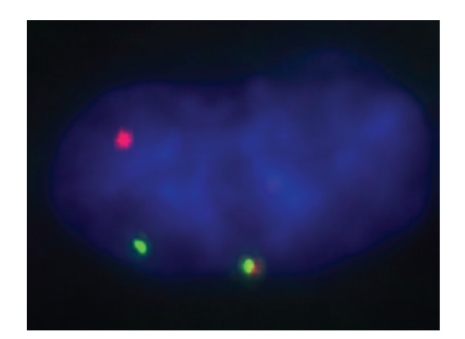
Photomicrograph of FISH break apart probe that targets the *ALK* gene. The normal chromosome (lower middle) shows a green signal and red signal in close proximity, whereas the green and red signals are far apart in the left aspect of the photomicrograph, confirming 1 copy of the *ALK* translocation.

Tumors other than IMTs have been recognized to demonstrate immunohistochemical expression of ALK, so genetic confirmation of the ALK translocation may be needed in problematic cases. At our institution, we find break-apart FISH testing to be quite useful in this setting (Figure 4). It should be noted, however, that a very recent report of response to the ALK inhibitor crizotinib in one patient with confirmed *ALK* translocation and no response in an IMT without *ALK* rearrangement [26] provides preliminary justification for FISH testing on all suspected IMTs. Secondary resistance to crizotinib in an IMT with ALK translocation (*ALK/RANBP2*) has been recently documented in another patient. This resistance was suspected to occur via the neuroblastoma-associated F1174L *ALK* mutation that has been well studied in neuroblastomas as a mechanism of resistance to some ALK inhibitors [27].

*ALK* perturbations in IMTs occur via fusion of the C-terminal kinase domain of anaplastic lymphoma kinase (*ALK*) gene located on 2p23. The *ALK* gene encodes a tyrosine kinase receptor that is normally only expressed in the developing nervous system [28]. In IMTs, the *ALK* gene fusion partner is most commonly tropomyosin 3 (*TPM3/ALK*) or tropomyosin 4 (*TPM4/ALK*), leading to constitutive activation. Other reported fusion partners include *CLTC*, *ATIC, RANBP2, CARS*, and *SEC31L1 *(Table 1). ALK along with its fusion partner tends to localize to the cytoplasm, but ALK/RANBP2 localizes to the nuclear membrane [29]. ALK function is poorly characterized at this time, so it is difficult to postulate the exact mechanism of oncogenesis; nevertheless, these fusions clearly lead to a survival and growth advantage to the cells harboring the translocation.

## 4. Inflammatory Fibroid Polyp (IFP)

**Figure 5:**
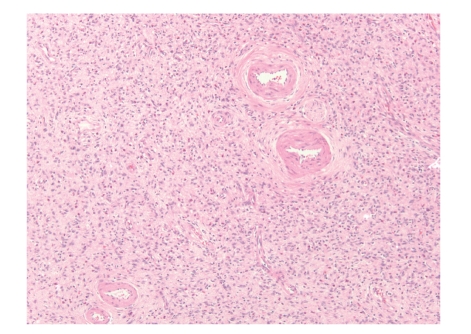
Photomicrograph of H&E-stained section from an inflammatory fibroid polyp. The lesion is moderately cellular with concentric whorls of spindle cells around blood vessels and numerous interspersed eosinophils.

IFPs are most often encountered in the stomach, usually presenting in the antrum, but are found throughout the GI tract. IFPs tend to arise within the submucosa and frequently extend into the overlying mucosa. These tumors are composed of bland spindle cells that often form perivascular cuffs (Figure 5). The tumor cells are embedded in a distinctive, granulation-type or fibromyxoid stroma with abundant capillary-type vessels. Characteristically, IFPs show a prominent eosinophilic infiltrate, but other inflammatory cells such as lymphocytes, mast cells, plasma cells, and histiocytes are encountered [1]. Given their gastrointestinal location, overlapping molecular features, and characteristic CD34 immunoreactivity, IFPs may be confused with GISTs [17]. Importantly, IFPs do not express other GIST-specific markers such as KIT or DOG-1 [36]. A few patients with germline PDGFRA mutations have been reported in the literature. The reports tend to consider these patients within the spectrum of familial GIST, but they present with a variety of tumors including GIST, GI neurofibromas, lipomas, and IFP-like polyps, some of which demonstrate a lipomatous stroma [12, 37, 38]. 

IFPs are rare benign tumors of uncertain histogenesis and were considered reactive processes, but recently, mutations in platelet-derived growth factor receptor alpha (*PDGFRA, *chromosome 4q12) were described in IFPs located in the stomach and small bowel [39]. In the stomach, 16/23 (70%) IFPs harbored activating mutations of *PDGFRA *[40]. Six of the mutations were located in exon 12, and 10 were located in exon 18. In the small bowel series, 33/60 (55%) harbored mutations in *PDGFRA*, 31 of which were located in exon 12, and 2 were in exon 18 [41]. Most of these mutations had been previously described in GIST. Extrapolating from the GIST literature, 41 of the 49 mutations detected in these series have been shown to cause at least in vitro ligand-independent activation. In addition, 100% of the stomach IFPs and 95% of small bowel IFPs showed expression of PDGFRA detected by immunohistochemistry, leading to the hypothesis that the cell of origin is a PDGFRA-positive mesenchymal cell [41]. 

**Figure 6:**
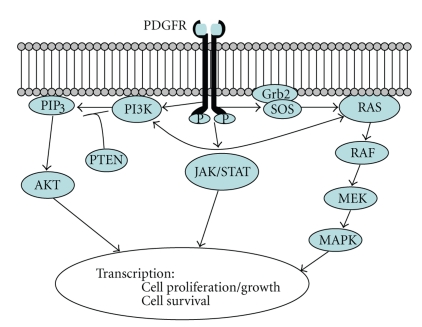
Schematic of platelet-derived growth factor receptor- (PDGFR-)related signaling pathways. Mutations can lead to activation of PDGFR independent of ligand binding. Numerous downstream pathways may lead to neoplastic advantages such as cell proliferation, growth, and survival.

PDGFRA is a receptor tyrosine kinase that is highly homologous to KIT [41]. Activation is normally ligand-dependent, but activating mutations cause constitutive activation (Figure 6). Ligand binding can cause homo-dimerization with another PDGFRA or hetero-dimerization with a PDGFR-beta. PDGFRA's interaction with several signaling pathways such as RAS/MAPK, PI3K, and JAK/STAT allows for the acquisition of numerous tumorigenic cell functions such as cell growth, migration, inhibition of apoptosis, and angiogenesis [42] when the receptor is constantly activated. The utility of *PDGFRA* mutation testing to confirm the diagnosis of IFP is minimal, but it is important to realize that not all *PDGFRA*-mutated mesenchymal neoplasms in the GI tract are GISTs.

## 5. Clear Cell Sarcoma (CCS)

Primary GI CCSs are extremely rare, with only about 20 total cases reported in the literature. Age range is quite variable, with the youngest diagnosed at age 13 years and the oldest at 85 [43]. The small bowel is most frequently involved, but reports of gastric, colonic, and mesenteric tumors also exist. These tumors are often metastatic to peritoneum, lymph nodes, or liver at presentation and are at significant risk for local recurrence after surgery [43]. Morphologically and immunophenotypically, there appear to be two CCS-like malignancies that occur in the GI tract. 

**Figure 7:**
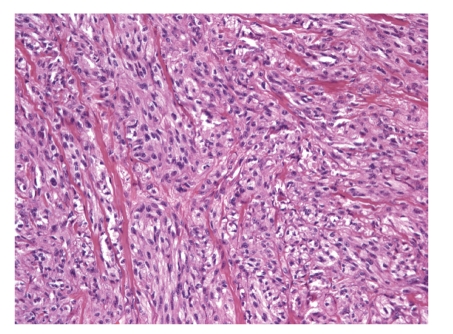
Photomicrograph of H&E-stained section from a conventional clear cell sarcoma. The cells are relatively bland with variably prominent nucleoli, low mitotic activity, and pale to clear cytoplasm. Fibrous bands illicit a compartmentalized appearance.

The first type of GI CCS constitutes those tumors that are morphologically, ultrastructurally, and immunohistochemically indistinguishable from CCSs of soft tissue. CCSs are typically composed of nests and fascicles of pale spindled to epithelioid cells, separated by delicate fibrous septa, forming a lobulated or organoid growth pattern (Figure 7). Cellular pleomorphism is typically uncommon, and multinucleated giant cells may be present. Nucleoli are typically prominent. Immunohistochemically, these tumors are indistinguishable from melanoma, being S-100 protein as well as melanocytic markers such as HMB-45 and Melan-A positive in the majority of cases [43]. In the soft tissue, these tumors are often referred to as “melanoma of soft parts,” because of their morphologic and immunohistochemical resemblance to malignant melanoma. Unfortunately, immunohistochemistry is of no help distinguishing the two malignancies, but the characteristic growth pattern and bland cytologic features with pale cytoplasm are useful clues to the diagnosis. As discussed below, rearrangements in the *EWRS1* gene occur in CCS, not melanoma, which can be invaluable in separating CCS from melanoma [44].

The second tumor type encountered in the GI tract is characterized by mostly epithelioid cells with pale cytoplasm without the characteristic tumor cell nesting or compartmentalization of soft tissue CCS. Osteoclast-like multinucleate giant cells are by definition admixed with the tumor cells such that the tumor has been described as “Osteoclast-rich tumor of the GI tract resembling CCS of soft parts.” The tumor cells reveal S-100 protein expression but lack any ultrastructural or immunohistochemical evidence of melanocytic differentiation. Other important morphologic differences with conventional CCS of soft tissue include indistinct nucleoli, more mitotic activity, and prominent cellular pleomorphism [43]. 

More than 90% of CCS of soft tissue are associated with the reciprocal translocation t(12;22)(q13;q12), which results in fusion of the *EWSR1* gene and the *ATF1* gene. This translocation links the N-terminal domain of *EWSR1* to the basic leucine zipper of *ATF1*. [43] Four fusion transcripts (Table 2) have been described in soft tissue tumors, the most common, or type 1, is a fusion of exon 8 of *EWSR1* and exon 4 of *ATF1* [45]. This translocation thus far has been the only described *EWSR1/ATF1* translocation in CCS of the GI tract. Another *EWSR1* translocation, *EWSR1/CREB1*, representing the t(2;22)(q34;q12) translocation, recently has been identified in a subset of GI and soft tissue CCS [46–48]. 

CREB1 and ATF1 belong to the basic leucine zipper superfamily of basic leucine zipper transcription factors. In normal melanocytes, CREB and ATF1 are involved in driving melanocytic differentiation. Both EWSR1/ATF1 and EWSR1/CREB1 fusion transcripts retain the basic leucine zipper domain, which mediates DNA binding and dimerization. In *EWSR1/ATF1* translocations, the activating domain of EWSR1 replaces the kinase inducible domain of ATF1, and the protein product has been shown to bind to microphthalmia-associated transcription factor, which in turn activates melanocyte stimulating hormone [29]. Overexpression of CREB contributes to metastatic potential in melanoma cells and is oncogenic in myeloid lines [46]. Genotype-phenotype correlation is imperfect in these GI tract CCS-like tumors such that either the *EWSR1/ATF1* or *EWSR1/CREB1* may occur in either tumor morphology described above [46].

**Figure 8:**
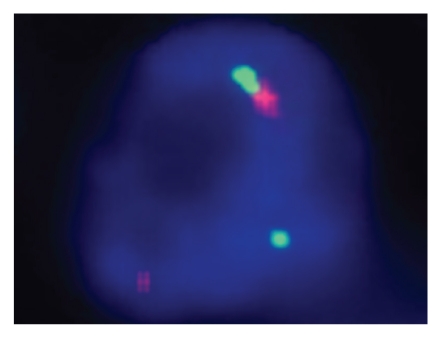
Photomicrograph of FISH break apart probe that targets the *EWSR1* gene. The normal chromosome (upper middle) shows a green signal and red signal in close proximity, whereas the green and red signals are far apart in the lower aspect of the photomicrograph, confirming *EWSR1* translocation.

Due to the rarity of CCS cases occurring in the GI tract, the first hurdle is considering the diagnosis. RT-PCR or FISH analyses are critical to the diagnosis of CCS, given its morphologic and immunophenotypic overlap with melanoma (Figure 8). It remains to be determined whether these S-100 protein positive tumors harboring *EWSR1* translocations represent a clinical, morphologic, immunophenotype, and genetic spectrum of one tumor [46] or are two distinct tumors [43].

## 6. Synovial Sarcoma (SS)

Primary GI SSs are quite uncommon. The Armed Forces Institute of Pathology (AFIP) reported a series of 10 SSs after undertaking a 30-year review of stomach mesenchymal neoplasms from 3 large centers. Prior to this series, there were 7 total reports of SS, 6 of which involved the esophagus, 1 involved the stomach, and all were biphasic. The AFIP series found 4 males and 6 females with an age range of 29–68 years. Two of the 8 patients with adequate followup died of their disease and two more showed local recurrence. Most tumors form a plaque-like mucosal mass and show uniform spindle cells in a haphazard arrangement with a collagenous background. Calcification or osseous metaplasia may be present. Nine of the 10 cases were of the monophasic type with one showing a poorly differentiated round cell component with high mitotic activity. The final case was biphasic, demonstrating a mixture of epithelial and spindled components. Mitotic activity is variable and can be greater than 50 per 10 HPF. All 7 tumors tested in the AFIP series showed the character *SYT/SSX* fusion transcripts (3 with *SYT/SSX1* and 4 with *SYT/SSX2*) [49]. 

The characteristic molecular alteration in SS is the t(X;18)(p11;q11) translocation, which usually fuses *SYT* on chromosome 18 with *SSX1* or *SSX2* on the X chromosome. Other less common fusion partners include *SSX4*, also located on the X chromosome. An *SYT* homolog, *SS18L1*, located on chromosome 20 also has been described as a fusion partner of *SSX1* in SS [50]. The functions of these gene products are unknown, and the oncogenic effects of the *SYT/SSX* fusion protein are unclear. Other genetic events may be necessary for sarcomagenesis and other molecular alterations have been described, including ERBB2 expression, IGF2 upregulation, CD44 repression, PTEN inactivation, and mutations associated with the wnt pathway [28]. Break-apart FISH probes are invaluable at our institution in confirming the diagnosis of SS, particularly in monophasic fibrous types, but PCR-based assays are used effectively at other institutions [50].

## 7. Smooth Muscle Tumors

**Figure 9:**
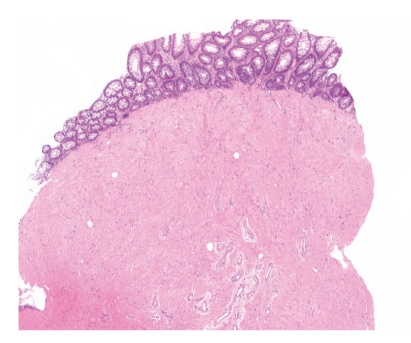
Photomicrograph of H&E-stained section from a leiomyomatous polyp. The leiomyoma is composed of bland smooth muscle cells with eosinophilic cytoplasm. The tumor is directly apposed to the colonic mucosa, suggesting derivation from the muscularis mucosae.

Leiomyomas of the GI tract show a male predominance and are most common in the colon and rectum, where they actually outnumber GIST [1]. The vast majority of GI leiomyomas are benign smooth muscle proliferations arising from the muscularis mucosae, often giving a polypoid endoscopic appearance (Figure 9) [51]. Rarely, cytologic atypia (“symplastic” leiomyoma) or even more rarely, mitotic activity may occur [18]. The second most common location for GI leiomyoma is in the distal esophagus, where they also outnumber GIST [51]. Esophageal leiomyomas usually are intramural in location, arising in the muscularis propria. Esophageal leiomyomas often show undulating borders and can range in size from <1 mm to >10 cm [1]. Various X chromosomal abnormalities, including collagen type IV alpha 5 and alpha 6, have been described in esophageal leiomyomas, which potentially accounts for the male predominance in these neoplasms [18]. GI leiomyomas may be confused with GISTs, and immunohistochemistry in this differential diagnosis can be quite helpful. Leiomyomas are invariably strongly positive for desmin, but are negative for CD117. One must not confuse CD117-positive mast cells that may be seen in between smooth muscles cells from true CD117 immunoreactivity in the smooth muscle cells [52].

GI leiomyosarcomas are quite rare [18, 53]. They are generally large masses and are characterized by cytologic atypia, high mitotic rate, and necrosis. Extra-intestinal leiomyosarcoma may show complex cytogenetic abnormalities [29] and have been recently shown to have loss of chromosomes 10q and 13q along with amplification of 17p13.1 to 11.2 [54], but the molecular pathology of GI leiomyosarcoma is unknown.

## 8. Schwannoma

**Figure 10:**
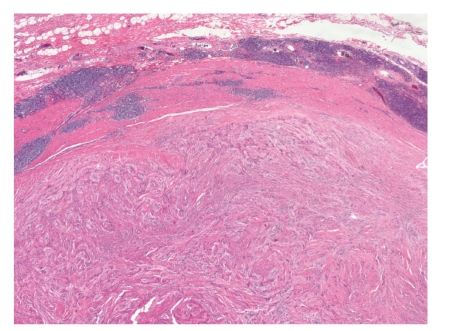
Photomicrograph of H&E-stained section from a gastric schwannoma. These tumors are often more easily recognized by their prominent lymphoid reaction at the periphery and lack of encapsulation rather than classic soft tissue schwannoma features such as Verocay bodies or thick, hyalinized vessels.

Schwannomas are most commonly encountered in the stomach [55] but are found throughout the GI tract [56, 57]. They may present as polyps or intramural masses. Their histologic appearance is often somewhat different than their extra-GI counterparts (Figure 10). GI schwannomas usually lack well-developed nuclear palisading, hyalinized vessel walls, or a capsule. Only rarely are the characteristic Antoni A and B patterns well developed, and Verocay bodies usually are not encountered. Distinguishing most schwannomas requires observation of the typical “wavy” nuclei with tapered ends; however, a minority may demonstrate an epithelioid morphology [57]. Regardless of morphology, all tumors demonstrate diffuse S-100 protein immunoreactivity while lacking CD117 positivity. Another characteristic feature of GI schwannomas is a prominent lymphoid infiltrate at the tumor periphery. GI schwannomas are benign and do not seem to occur in the setting of NF2 [1]. In addition, GI schwannomas show no *NF2* mutations and only rare LOH of ch. 22q [56], which is different than their soft tissue counterparts. Rare examples tested for *KIT* mutations have been wild type [58].

## 9. Mesenchymal Polyps

A variety of mesenchymal lesions present as incidentally identified polyps during routine endoscopic procedures (usually colonoscopy), including perineuriomas/fibroblastic polyps [59], muscularis mucosae leiomyomas [51], Schwann cell “hamartomas,” [60], granular cell tumors [61], elastofibromatous polyps [62], and ganglioneuromas [63]. Colonic perineuriomas/fibroblastic polyps are often associated with a hyperplastic polyp-like component with serrated epithelial crypts. A recent study reported BRAF mutations in 63% of the epithelial component of these polyps. The authors concluded that the polyps are true mixed epithelial-mesenchymal neoplasms, but there remains no conclusive evidence that the spindle cell proliferation is truly neoplastic [64]. Ganglioneuromas are benign lesions composed of ganglion cells, nerve fibers, and Schwann cells [60] that often arise in the setting of inherited tumor syndromes, including Cowden syndrome (PTEN mutations), MEN 2B, and NF1 [60], among others. Solitary ganglioneuromas are not considered to be a marker of an inherited syndrome. Aside from syndrome-associated ganglioneuromas, the molecular pathologic aspects of these benign polypoid mesenchymal lesions are uncharacterized.

## 10. Adipocytic Tumors

**Figure 11:**
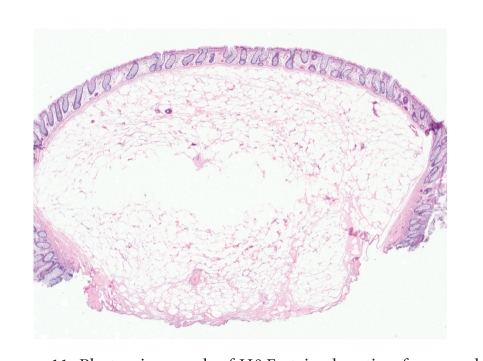
Photomicrograph of H&E-stained section from a submucosal lipoma in the colon. The tumor is composed of mature fibroadipose tissue with sharp demarcation from the overlying mucosa.

Lipomas are most common in the right colon and usually are identified as small intramural polypoid lesions. They are usually centered within the submucosa and composed of mature-appearing adipocytes that are relatively uniform in size and lack cytologic atypia (Figure 11). These lesions are often endoscopically suspected after eliciting the “pillow sign” with closed biopsy forceps [65]. No molecular characterization of GI lipomas exists; conventional soft tissue lipomas exhibit abnormal karyotypes in about 60% of cases, most commonly involving rearrangement of chromosome 12q13~15 encompassing the chromatin remodeling gene *HGMA2 *[66].

Primary GI liposarcomas are exceptionally rare tumors. Most liposarcomas that involve the gut arise within the retroperitoneum, and this is one of the more common causes of a sarcoma presenting as a GI mass. Typical well-differentiated liposarcomas are fatty tumors that demonstrate large, atypical cells embedded within fibrous septa or between the fat cells, but other morphologies such as inflammatory or sclerosing exist. Conventional liposarcoma may de-differentiate and at least partially loose their typical well-differentiated component. About 80% of well-differentiated liposarcomas are characterized by ring or giant marker chromosomes derived from chromosome 12q13~q15, including *MDM2, HGMA2*, and other genes [67]. When more sensitive methods such as FISH, PCR, or immunohistochemistry are employed, >95% of tumors show MDM2 amplification [68, 69]. Therefore, assessing for amplification of *MDM2* by FISH or immunohistochemistry is a powerful tool in supporting the diagnosis liposarcoma secondarily involving the GI tract.

## 11. Glomus Tumors

These tumors are morphologically similar to their counterparts that are most commonly encountered in the distal extremities. The vast majority of GI glomus tumors have been documented to occur in the stomach with fewer occurring in the intestines and <150 cases have been reported in the English literature [70]. Glomus tumors are composed of a proliferation of sharply demarcated modified smooth muscle cells, which are often arranged around dilated staghorn vessels. The cells contain a round nucleus and pale cytoplasm and generally low mitotic activity. Focal atypia and vascular invasion reportedly are common. In the AFIP series of 32 gastric glomus tumors, none of the 5 tumors tested for mutations in exons 9 or 11 of the KIT gene showed a mutation [70]. Otherwise, the molecular pathology of glomus tumors is unknown.

## 12. Conclusion

Primary mesenchymal tumors of the GI tract are rare, but like their soft tissue counterparts, molecular pathology often plays a critical role in the work-up of these tumors. Molecular pathology plays a major role the diagnosis of these tumors, particularly in clear cell sarcoma, inflammatory myofibroblastic tumor, synovial sarcoma, and liposarcoma, among others. Powerful prognostic data also is emerging such as in inflammatory myofibroblastic tumor with *ALK* rearrangements and possibly *CTNNB1* mutations in desmoids. The future of molecular pathology is in predictive molecular testing—molecular pathology tests aimed at aiding our clinical colleagues in selecting the best treatments for our patients. Although personalized therapy is not standard of care yet in these rare tumors, the recent ALK antagonist case reports in IMTs suggest that it is only a matter of time.

## Figures and Tables

**Table 1:** Summary of various ALK fusions in IMT.

Fusion partner	Chromosomal location	ALK staining location (Gleason 2008)
*TPM3* [30]	1q22-23	Cytoplasmic
*TPM4* [30]	19p13.1	Cytoplasmic
*CARS* [31]	11p15	Cytoplasmic
*ATIC* [32]	2q35	Cytoplasmic
*SEC31L1* [33]	4q21	Cytoplasmic
*RANBP2* [34]	2q13	Nuclear membrane
*CLTC* [35]	17q23	Granular cytoplasmic

**Table 2:** Summary of various EWSR1 fusions in CCS [45, 46].

*EWSR1/ATF1* transcript	Relative frequency	GI tract
*EWSR1* exon 8/ *ATF1* exon 4	common	Yes
*EWSR1* exon 7/ *ATF1* exon 5	common	No
*EWSR1* exon 10/ *ATF1* exon 5	uncommon	No
*EWSR1* exon 7 *ATF1* exon 7	uncommon	No
*EWSR1* exon 7/ *CREB1* exon 7	uncommon	Yes
